# Metabolic dysregulation and cancer mortality in a national cohort of blacks and whites

**DOI:** 10.1186/s12885-017-3807-2

**Published:** 2017-12-15

**Authors:** Tomi Akinyemiju, Justin Xavier Moore, Suzanne Judd, Susan Lakoski, Michael Goodman, Monika M. Safford, Maria Pisu

**Affiliations:** 1 Departments of Epidemiology, University of Alabama at Birmingham, Birmingham, AL USA; 2 Comprehensive Cancer Center, University of Alabama at Birmingham, Birmingham, AL USA; 3 Department of Epidemiology, University of Kentucky, Lexington, KY USA; 4 Department of Emergency Medicine, University of Alabama at Birmingham, Birmingham, AL USA; 5 Department of Biostatistics, University of Alabama at Birmingham, Birmingham, AL USA; 6 Division of Clinical Cancer Prevention, The University of Texas MD Anderson Cancer Center, Houston, TX USA; 7 Department of Epidemiology, Emory University School of Public Health, Atlanta, GA USA; 8 Division of General Internal Medicine, Weill Cornell Medical College, New York, NY USA; 9 Division of Preventive Medicine, University of Alabama at Birmingham, Birmingham, AL USA

**Keywords:** Metabolic syndrome, Cancer, Racial disparities, Cancer mortality, Survival

## Abstract

**Background:**

We examined the association between metabolic dysregulation and cancer mortality in a prospective cohort of Black and White adults.

**Methods:**

A total of 25,038 Black and White adults were included in the analysis. Metabolic dysregulation was defined in two ways: 1) using the joint harmonized criteria for metabolic syndrome (MetS) and 2) based on factor analysis of 15 variables characterizing metabolic dysregulation. We estimated hazards ratios (HRs) and 95% confidence intervals (CIs) for the association of MetS and metabolic dysregulation with cancer mortality during follow-up using Cox proportional hazards models.

**Results:**

About 46% of Black and 39% of White participants met the criteria for MetS. Overall, participants with MetS (HR: 1.22, 95% CI: 1.03–1.45) were at increased risk of cancer-related death. In race-stratified analysis, Black participants with MetS had significantly increased risk of cancer mortality compared with those without MetS (HR: 1.32, 95% CI: 1.01–1.72), increasing to more than a 2-fold risk of cancer mortality among those with five metabolic syndrome components (HR: 2.35, 95% CI: 1.01–5.51).

**Conclusions:**

There are marked racial differences in the prevalence of metabolic dysregulation defined as MetS based on the harmonized criteria. The strong positive associations between MetS and cancer mortality suggests that efforts to improve cancer outcomes in general, and racial disparities in cancer outcomes specifically, may benefit from prevention and management of MetS and its components.

**Electronic supplementary material:**

The online version of this article (10.1186/s12885-017-3807-2) contains supplementary material, which is available to authorized users.

## Background

Significant racial disparities in cancer mortality have been well documented in the United States (US), with at least a 13% difference in 5-year survival observed between Blacks and Whites diagnosed with breast and colorectal cancer [1]. Black adults are more likely to develop metabolic dysregulation and meet the formal criteria for metabolic syndrome (MetS). MetS is a cluster of interrelated biochemical abnormalities that include central obesity, insulin resistance, dyslipidemia and hypertension, and has been shown to significantly increase the risk of coronary heart disease, stroke and type-2 diabetes [2–5]. Individual components of MetS, specifically obesity [6, 7], diabetes [8], and hypertension [9, 10], have been associated with increased risk for cancer; however, the entire cluster of MetS components has only more recently been shown to be associated with cancer risk and outcome.

Several studies show a significant and independent positive association between MetS and cancer incidence [11–13], distant metastasis [14] and aggressive cancer phenotypes [15–17]. A recent systematic review and meta-analysis of 43 studies found significant associations between MetS and risk of liver cancer, colorectal cancer, and bladder cancer [18]. Importantly, the association between MetS and cancer risk has consistently been found to be larger than the corresponding associations for individual MetS components, suggesting that there may be independent, complex biological processes underlying this association. However, despite renewed interest in the role of MetS in cancer etiology and outcomes, many of the prior studies have suffered from significant lead-time, length and selection biases, with only a few studies examining racial differences in this association. Few prospective studies have examined objective measures of MetS at baseline in relation to cancer mortality, with racially diverse study populations to assess race-specific risk. Furthermore, although there is growing consensus regarding the biological importance of MetS, questions remain about the clinical definition, categorical cut-points and included components, especially in relation to cancer risk. In this study, we assess MetS and variables associated with metabolic dysregulation at baseline among Blacks and Whites, and examine the association with cancer mortality during follow-up.

This study takes advantage of the large REasons for Geographic and Racial Differences in Stroke (REGARDS) cohort, with individual assessment of MetS components at baseline, and cancer mortality identified prospectively during follow-up. The objective of the current study was to determine the association between MetS and metabolic dysregulation with cancer mortality among Black and White adults in the US.

## Methods

### Data source

REGARDS is one of the largest ongoing national longitudinal cohorts of community-dwelling adults in the US [19]. Designed to examine factors contributing to racial and geographic differences in stroke mortality, the REGARDS study includes 30,239 participants ages 45 years and older at baseline; 45% male, 41% Black, and 69% >60 years old [19]. REGARDS participants were randomly sampled from all US states and recruited via mail and telephone. REGARDS was originally designed to evaluate risk factors and racial disparities in stroke, therefore 30% of participants were recruited from the ‘Stroke Belt’ (Alabama, Arkansas, Georgia, Louisiana, Mississippi, North Carolina, South Carolina, and Tennessee*),* 20% from the ‘Stroke Buckle’ (Georgia, North Carolina, and South Carolina*)* and approximately 50% from other US states. Participants were recruited between January 2003 and October 2007, and detailed information about demographics, health behaviors, chronic medical conditions, physical status, diet, and medications were collected. In addition, baseline in-home visits were scheduled for all enrolled patients, during which anthropometric data including body weight, waist circumference, height, and blood pressure were obtained. Participants were asked to fast overnight for 10–12 h before visitation and to have medications available during the time of visitation. Trained technicians collected blood and urine samples and assessed current medication use. Participants were subsequently contacted by telephone every 6-months to identify medical events or hospitalizations experienced since the prior contact. Medical events or deaths were ascertained using death certificates, medical records, and/or interviewed proxies to determine causes of the death.

### Main exposure variables

We defined MetS based on the recently published consensus statement developed by multiple health and professional organizations [20]. The joint harmonized criteria by Alberti et al. defined MetS as the presence of at least three of: 1) Diabetes: fasting glucose ≥126 mg per liter (mg/L) (or a glucose ≥200 mg/L for those not fasting) or the use of insulin or oral hypoglycemic agents; 2) High triglycerides: triglycerides ≥150 mg per deciliter (mg/dL) or reported use of medication for elevated triglycerides; 3) Dyslipidemia: low high-density lipoprotein (HDL) cholesterol <40 mg/dL for males and <50 mg/dL for females, or use of lipid lowering medications; 4) Hypertension: systolic blood pressure ≥ 140 mmHg, diastolic blood pressure ≥ 90 mmHg, or the reported use of antihypertensive agents; and 5) Obesity: increased waist circumference (WC) >102 cm for males or >88 cm for females.

### Secondary exposure variable

We additionally defined metabolic dysregulation based on factor analysis of 15 metabolic related variables (height, weight, WC, body mass index (BMI), log of triglycerides, cholesterol, HDL cholesterol, log of low-density lipoprotein (LDL) cholesterol, dyslipidemia, systolic blood pressure, diastolic blood pressure, hypertension, log of insulin, glucose, and diabetes). We used orthogonal rotation of each component in the factor analysis, examined the scree plots and employed an eigenvalue cut-point of approximately 1.0 for inclusion of final derived factors. We calculated final factor loadings of 6 factors based on the full sample, and presented results only for factors with absolute values for loadings >0.4. We named patterns based on the factor loadings that contributed most highly to each pattern (Additional file 1). For instance, Factor 1 was termed “Obesity” since it was loaded heavily by variables associated with body weight such as weight (0.91), waist circumference (0.86), BMI (0.94), and log insulin (0.61). Similarly, Factor 4 was termed “Lipids” since it was loaded heavily by log triglycerides (0.76), HDL cholesterol (−0.69), and dyslipidemia (0.70).

### Cancer mortality

Cancer mortality was identified through semi-annual telephone follow-up, death information from participant proxies, linkages with the Social Security Death Index (SSDI) as well as the National Death Index (NDI). Date of death was confirmed using death certificates, SSDI and/or NDI, and cause of death was adjudicated by a committee of experts using all available information as recommended by national guidelines [21]. Follow-up data for this analysis was available through December 31, 2012.

### Participant characteristics

Demographic information used for analysis included age, race, gender, income, education, and geographic location. Health behaviors included tobacco and alcohol use. Chronic medical conditions assessed included: atrial fibrillation, chronic lung disease, chronic kidney disease, coronary artery disease, deep vein thrombosis, diabetes, dyslipidemia, hypertension, myocardial infarction, obesity, peripheral artery disease, and stroke. In addition, we included serum high-sensitivity C-reactive protein (hsCRP) as a covariate, since multiple studies have demonstrated racial differences in CRP [22–24].

### Statistical analysis

We compared baseline characteristics by race using chi-square tests for categorical characteristics, analysis of variance (ANOVA) for continuous variables, and Kruskal-Wallis test for non-normal continuous variables. We categorized each metabolic factor into quartiles based on the distribution among study participants, with the highest quartile corresponding to participants at the highest distribution of the factor. Thus, participants in the fourth quartile of the cholesterol factor had the highest mean cholesterol levels in the study. To estimate the hazards of cancer mortality, we fit Cox proportional hazard models examining each MetS component independently and jointly in relation to cancer mortality. We a priori specified examination of race-stratified models, and stratified statistical models by race groups after adjusting for socio-demographics, health behaviors, baseline medical conditions, and hs-CRP. In sensitivity analysis we adjusted models for socio-demographics and health behaviors. The results of all models were expressed as adjusted hazard ratios (AHR) and the corresponding 95% confidence intervals (CI). Individuals were censored at the time of death, loss to follow-up, or the end of cancer mortality ascertainment (December 31, 2012). SAS version 9.4 and STATA version 13 were used for all statistical analysis. We considered two-sided *p* values <0.05 as statistically significant.

## Results

### Cohort characteristics

**Fig. 1:**
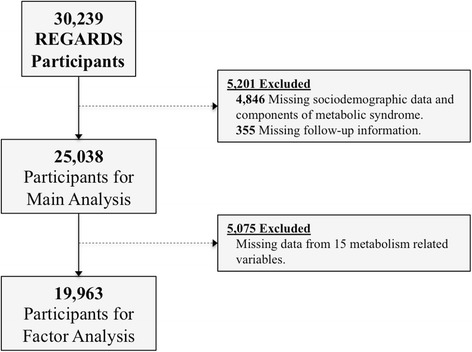
Study flowchart and breakdown of study participants used in analyses

Among 30,239 REGARDS participants, 5201 were excluded due to missing data for exposure (components of metabolic syndrome) or follow-up time, resulting in 25,038 participants remaining for the main analysis (Fig. 1). The most common cancer deaths were lung (27.5%), gastro-intestinal (20.6%), and hematological (10.6%) (Additional file 1). As shown in Table 1, Black participants were younger (63.8 vs. 65.3 years), less likely to be male (37.9% vs. 49.9%), had lower education (19.0% vs. 7.1% with less than high school education), and lower income (25.6% vs. 11.8% with less than $20,000 household income) compared with White participants (*p* values <0.01). Compared with White participants, Black participants had higher total body weight (87.7 vs. 82.4 kg), WC (97.9 vs. 94.8 cm), BMI (30.7 vs. 28.3 kg per meter squared [kg/m^2^]), and LDL-cholesterol (115.0 vs. 110.0 mg/dL), but had lower levels of triglycerides (95.0 vs. 122 mg/dL), (*p* values <0.01). Although Black participants were less likely to have dyslipidemia (54.6% vs. 61.5%, *p* value < 0.02), they were more likely to have hypertension (71.0% vs. 50.3%) and diabetes (29.4% vs. 16.2%) compared with White participants (*p* value <0.01). The formal criteria for MetS was met among 45.8% of Black participants and 38.8% of White participants (*p* value <0.01).

**Table 1:** Baseline characteristics of REGARDS participants by race (*N =* 25,038)

	Black (*N =* 9822)	White (*N =* 15,216)	*p* value^a^
Age^b^	63.8 (9.3)	65.3 (9.4)	<0.01
Male Gender (%)	3721 (37.9)	7588 (49.9)	<0.01
≤ High School Education (%)	1864 (19.0)	1086 (7.1)	<0.01
≤ $20,000 Income (%)	2511 (25.6)	1796 (11.8)	<0.01
High WC/ Obesity			
Weight (kg)^b^	87.7 (20.1)	82.4 (18.9)	<0.01
Height (in.)^b^	66.4 (4.3)	67.0 (4.0)	<0.01
WC (cm)^b^	97.9 (15.2)	94.8 (15.5)	<0.01
BMI^b^	30.7 (6.7)	28.3 (5.6)	<0.01
Elevated Triglycerides			
Triglycerides (mg/dL)^c^	95.0 (72.0–130.0)	122.0 (88.0–174.0)	<0.01
Total Cholesterol (mg/dL)^b^	193.3 (40.9)	192.0 (39.5)	0.01
LDL-Cholesterol (mg/dL)^c^	115.0 (92.0–140.0)	110.0 (89.0–134.0)	<0.01
Dyslipidemia (%)	5325 (54.6)	9294 (61.5)	<0.01
Reduced HDL Cholesterol			
HDL-Cholesterol (mg/dL)^b^	53.7 (16.0)	50.8 (16.1)	<0.01
Elevated Blood Pressure			
DBP (mmHg)^b^	78.6 (10.1)	75.2 (9.2)	<0.01
SBP (mmHg)^b^	130.7 (17.3)	125.4 (15.9)	<0.01
Hypertension (%)	6965 (71.0)	7638 (50.3)	<0.01
Elevated Fasting Glucose			
Insulin (uU/mL)^c^	10.9 (6.8–17.2)	8.8 (5.6–13.8)	<0.01
Fasting glucose (mg/dL)^b^	107.0 (38.9)	99.4 (26.9)	<0.01
Diabetes (%)	2873 (29.4)	2460 (16.2)	<0.01
Metabolic Components ^d^(%)			
0	642 (6.5)	2193 (14.4)	<0.01
1	1910 (19.5)	3577 (23.5)	
2	2774 (28.2)	3532 (23.2)	
3	2615 (26.6)	2897 (19.0)	
4	1482 (15.1)	2041 (13.4)	
5	399 (4.1)	976 (6.4)	

^a^Significance determined using Chi-square test for categorical, ANOVA for continuous, or Kruskal-Wallis test for non-parametric continuous variables

^b^Presented as mean (standard deviation) for normal continuous characteristics

^c^Presented as median (interquartile range) for non-parametric continuous characteristics

^d^Metabolic components are high waist circumference (WC), elevated triglycerides, reduced HDL cholesterol, elevated blood pressure, and elevated fasting glucose

*BMI* Body Mass Index, *HDL* high-density lipoprotein, *LDL* low-density lipoprotein, *DBP* diastolic blood pressure, *SBP* systolic blood pressure

### Metabolic syndrome and cancer mortality

**Fig. 2:**
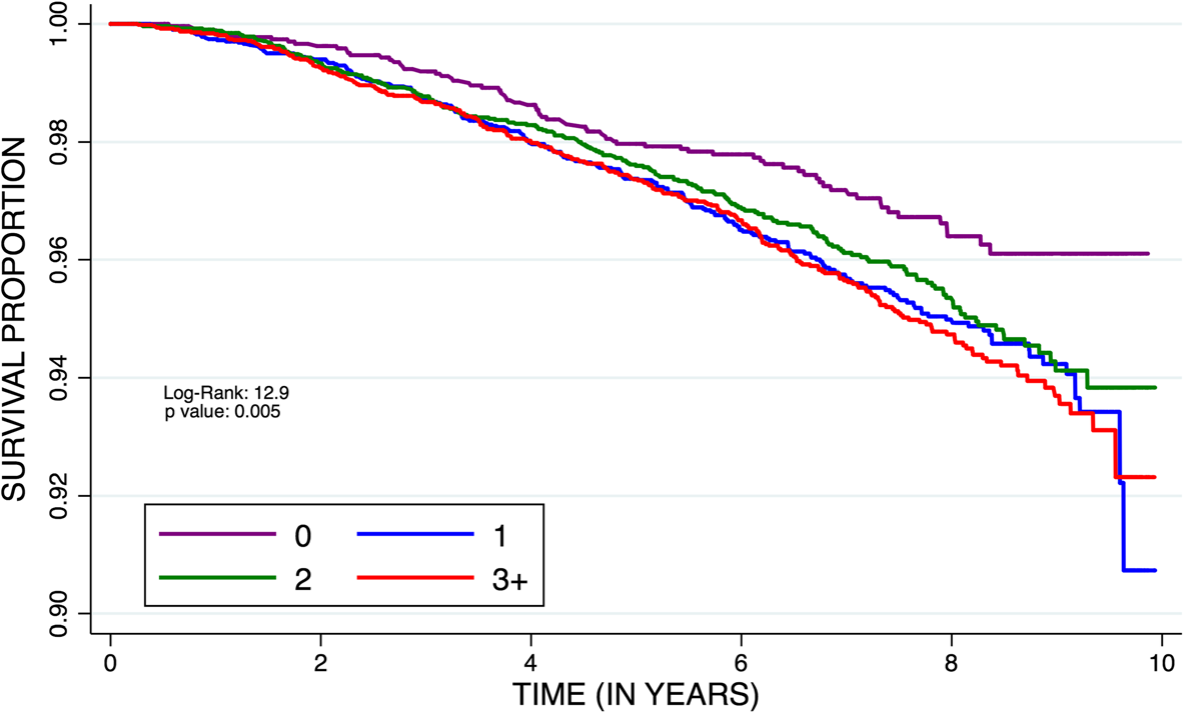
Kaplan-Meier plot for time to cancer death by number of metabolic syndrome components

Among all participants (Table 2), those with MetS were at increased risk of cancer mortality compared with those without MetS (AHR: 1.22; 95% CI: 1.03–1.45). The risk of cancer-related deaths increased with the number of MetS components; participants with three (AHR: 1.41, 95% CI: 1.01–1.97), four (AHR: 1.62, 95% CI: 1.12–2.34) or five (AHR: 1.59, 95% CI: 1.01–2.51) components experienced significantly increased risk of cancer mortality (Table 2; Fig. 2) compared with participants with none. Black participants with any three (AHR: 1.89, 95% CI: 1.02–3.49), four (2.44, 95% CI: 1.26–4.74) or five (2.35, 95% CI: 1.01–5.51) MetS components had a 2-fold increased risk of cancer mortality compared with those with none. However, there were no significant associations between MetS and risk of cancer morality among White Participants, and there was no evidence of a statistical interaction on the multiplicative scale between MetS components and race (p-values >0.05). Among Black participants, those with reduced HDL-cholesterol (AHR: 1.30, 95% CI: 1.02–1.64) and elevated fasting glucose (AHR: 1.40, 95% CI: 1.09–1.81) were at an increased risk of cancer mortality. Among White participants, those with reduced HDL-cholesterol (AHR: 1.27, 95% CI: 1.03–1.55) were at an increased risk of cancer mortality. The associations between MetS and cancer mortality attenuated in models excluding baseline chronic medical conditions (Additional file 2).

**Table 2:** Hazard ratios (HRs)^a^ and 95% confidence intervals for the association between metabolic syndrome (MetS) and cancer mortality

	HR (95% CI)			
	Black (*N* = 402)^b^	White (*N* = 595)^b^	All (*N* = 997)^b^	*p* value_interaction_^c^
Metabolic Syndrome	**1.32 (1.01–1.72)**	1.18 (0.94–1.48)	**1.22 (1.03–1.45)**	0.28
Components				
High WC	1.36 (0.75–2.46)	1.32 (0.73–2.36)	1.30 (0.86–1.96)	0.07
Elevated Triglycerides	0.96 (0.71–1.28)	1.04 (0.86–1.25)	1.00 (0.86–1.16)	0.91
Reduced HDL Cholesterol	**1.30 (1.02–1.64)**	**1.27 (1.03–1.55)**	**1.27 (1.09–1.48)**	0.27
Elevated blood pressure	1.43 (0.96–2.15)	0.97 (0.73–1.28)	1.11 (0.89–1.40)	0.89
Elevated fasting glucose	**1.40 (1.09–1.81)**	1.02 (0.83–1.26)	1.16 (0.99–1.36)	0.57
# Metabolic Syndrome Components				
0 (Referent)	Referent	Referent	Referent	0.98
1	1.28 (0.73–2.24)	1.09 (0.78–1.52)	1.15 (0.86–1.53)	
2	1.63 (0.92–2.88)	1.07 (0.74–1.54)	1.24 (0.91–1.68)	
3	**1.89 (1.02–3.49)**	1.23 (0.82–1.85)	**1.41 (1.01–1.97)**	
4	**2.44 (1.26–4.74)**	1.32 (0.84–2.08)	**1.62 (1.12–2.34)**	
5	**2.35 (1.01–5.51)**	1.36 (0.78–2.38)	**1.59 (1.01–2.51)**	

^a^ Analysis based on 25,038 REGARDS participants with non-missing data on exposure and covariates. Models adjusted for age, sex, race (all model only), education, region, income, tobacco use, alcohol use, and baseline chronic medical conditions, and hs-CRP

^b^ *N =* number of cancer death events

^c^ Interaction significance between race*factor (i.e., metabolic syndrome, high waist circumference (WC), and metabolic syndrome components) using Wald test

Bold indicates statistically significant at 0.05 alpha level

### Metabolic dysregulation and cancer mortality

We performed factor analysis and identified six distinct factors associated with metabolic dysregulation among 19,963 participants with complete data on 15 metabolism-related variables (Fig. 1 and Additional file 3, previously introduced in methods). The distribution of each metabolic dysregulation factor by demographic and socioeconomic characteristics of REGARDS participants are provided in Additional file 4. Among all participants (Table 3 and Additional file 5), those in the highest quartile of the glucose (4th quartile AHR: 1.35; 95% CI: 1.08–1.61) were at an increased risk of cancer mortality, while participants in the highest quartiles for obesity (4th quartile AHR: 0.68; 95% CI: 0.49–0.94) and cholesterol (3rd vs. 1st quartile AHR: 0.81; 95% CI: 0.65–0.99) factors had reduced risk of cancer mortality compared with those in the first quartile. Black and White participants within higher quartiles of the obesity factor had a reduced risk of cancer mortality compared with those in the lowest quartile, however Blacks in the highest quartile of the glucose factor experienced increased risk (AHR: 1.57; 95% CI: 1.10–2.23) for cancer mortality compared with those in the first quartile. White participants with one (AHR: 1.60, 95% CI: 1.16–2.19) or two (AHR: 1.52, 95% CI: 1.08–2.14) of any factor in the highest quartile were at an increased risk of cancer mortality. The associations between derived factors for metabolic dysregulation and cancer mortality were similar in models excluding baseline chronic medical conditions (Additional file 6).

**Table 3:** Hazard ratios (HRs) ^a^ and 95% confidence intervals (CIs) for the association between metabolic dysregulation factors and cancer mortality

	Black (*N* = 287) ^b^	White (*N* = 497) ^b^	All (*N* = 784) ^b^	*p* value_interaction_^c^
Obesity				
1st Quartile (Ref)	Referent	Referent	Referent	0.25
2nd Quartile	**0.69 (0.48–0.98)**	**0.77 (0.60–0.99)**	**0.77 (0.62–0.94)**	
3rd Quartile	**0.57 (0.37–0.88)**	**0.68 (0.50–0.94)**	**0.72 (0.56–0.93)**	
4th Quartile	0.63 (0.38–1.06)	0.71 (0.47–1.07)	0.84 (0.61–1.16)	
Cholesterol				
1st Quartile (Ref)	Referent	Referent	Referent	0.27
2nd Quartile	0.92 (0.66–1.28)	**0.68 (0.52–0.88)**	**0.77 (0.63–0.94)**	
3rd Quartile	0.76 (0.53–1.09)	0.83 (0.64–1.08)	**0.81 (0.65–0.99)**	
4th Quartile	0.87 (0.61–1.25)	0.86 (0.66–1.13)	0.86 (0.70–1.07)	
Blood Pressure				
1st Quartile (Ref)	Referent	Referent	Referent	0.55
2nd Quartile	1.02 (0.68–1.54)	1.06 (0.81–1.39)	1.06 (0.85–1.32)	
3rd Quartile	0.89 (0.57–1.39)	0.87 (0.63–1.21)	0.89 (0.69–1.16)	
4th Quartile	0.78 (0.48–1.25)	0.92 (0.64–1.31)	0.86 (0.65–1.14)	
Lipids				
1st Quartile (Ref)	Referent	Referent	Referent	0.55
2nd Quartile	1.28 (0.91–1.79)	0.95 (0.70–1.29)	1.07 (0.85–1.33)	
3rd Quartile	1.12 (0.73–1.74)	0.92 (0.64–1.33)	1.00 (0.76–1.32)	
4th Quartile	1.42 (0.86–2.32)	1.20 (0.81–1.79)	1.30 (0.96–1.77)	
Height				
1st Quartile (Ref)	Referent	Referent	Referent	0.07
2nd Quartile	0.90 (0.60–1.33)	1.00 (0.74–1.36)	0.97 (0.77–1.23)	
3rd Quartile	1.34 (0.89–2.03)	0.86 (0.61–1.23)	1.04 (0.80–1.36)	
4th Quartile	0.91 (0.56–1.49)	0.83 (0.56–1.22)	0.88 (0.65–1.19)	
Glucose				
1st Quartile (Ref)	Referent	Referent	Referent	0.58
2nd Quartile	1.28 (0.88–1.85)	1.20 (0.91–1.58)	1.23 (0.99–1.54)	
3rd Quartile	1.15 (0.78–1.68)	1.23 (0.93–1.62)	1.21 (0.97–1.51)	
4th Quartile	**1.57 (1.10–2.23)**	1.21 (0.90–1.62)	**1.35 (1.08–1.69)**	
# Factor Variables in 4th Quartile				
0 (Referent)	Referent	Referent	Referent	0.08
1	0.83 (0.56–1.21)	**1.60 (1.16–2.19)**	1.27 (0.99–1.62)	
2	0.92 (0.62–1.36)	**1.52 (1.08–2.14)**	1.25 (0.97–1.62)	
3+	1.02 (0.65–1.62)	1.39 (0.94–2.07)	1.23 (0.91–1.66)	

^a^Analysis based on 19,963 REGARDS participants with non-missing data on all factor analysis component variables. Models adjusted for age, sex, race (all model only), education, region, income, tobacco use, alcohol use, and baseline chronic medical conditions, and hs-CRP

^b^ *N =* number of cancer death events

^c^ Interaction significance between race*factor using Wald test. Bold indicates statistically significant at 0.05 alpha level

## Discussion

In a large prospective cohort of community-dwelling adults, we observed significant differences in the prevalence of MetS and metabolic dysregulation by race. This study fills a significant gap in the literature regarding the role of metabolic dysregulation, defined here using the harmonized MetS criteria as well as factor analysis, in relation to cancer mortality. Black participants in this study had a higher prevalence of MetS and dysregulated metabolic components when compared with White participants. Overall, participants who met the criteria for MetS were at significantly higher risk for cancer mortality, and the risk increased with increasing number of components. Of the factor analysis-derived metabolic components, elevated glucose levels was strongly associated with increased mortality, while obesity and high cholesterol were associated with reduced cancer mortality. In race-stratified analysis, Blacks with MetS had about a 2-fold increased risk of cancer mortality compared with those without MetS, however there were no significant associations among Whites.

The joint harmonized criteria for MetS was developed to generate consensus regarding the clinical importance and standardized definition of this condition[20], further stimulating renewed attention into its impact on health outcomes. The link between individual metabolic components, i.e. central obesity, insulin resistance, dyslipidemia and chronic diseases have been known for many decades, although only recently has compelling research studies begun to emerge on the biological consequence the MetS cluster in chronic diseases. Metabolic syndrome has been associated with significantly increased risk of coronary heart disease and stroke in multiple research studies [2–5]. Furthermore, individual components of MetS – specifically obesity [6, 7], diabetes [8], and hypertension [9, 10] – have also been associated with increased risk of multiple cancers, and MetS has been shown to be as a strong risk factor for breast cancer, with odds ratios ranging from 2.50 in Brazil, (95% CI: 1.17–5.30) [11], to 6.28 in Italy (95% CI 2.79–14.11) [13]. Strikingly, women with MetS who develop breast cancer were more than twice as likely to develop distant metastasis [14], and aggressive tumors [16, 17]. What has remained less controversial is the inextricable link between poor diet and obesity with MetS, and an understanding that these predisposing factors are more prevalent among Blacks compared with Whites- a trend that was observed in this study and has remained consistent for several decades [25, 26].

The consistent associations observed in this study between having MetS and increased risk of cancer mortality, as well as higher risk with increasing number of MetS components suggests that prevention strategies focused on these modifiable factors, i.e. obesity, cholesterol, blood pressure and glucose, are warranted and should be considered as part of comprehensive cancer control and prevention plans. Unfortunately, past strategies to reduce the prevalence of MetS risk factors may have had limited success due to individual and community level factors such as poverty, lack of availability of fresh food, safe walking environments and routine access to preventive care in many communities [27, 28]. More research will be needed to better identify race-specific public health strategies that have the best chance of eliminating individual MetS components as well as clinical strategies to control the entire cluster of MetS, since those strategies may have significant potential to reduce racial disparities in cancer mortality.

Certain limitations are relevant to the interpretation of this study. First, although metabolic factors were measured at baseline prior to cancer diagnosis or mortality, observed values may have been subject to information biases. Second, although we adjusted for confounding due to several baseline covariates, physical activity variables that may affect lipid and blood pressure levels were not included in the analysis. In addition, the total number of cancer deaths may be underestimated in this population as the REGARDS study was primarily intended to identify incident stroke events was not specifically focused on cancer outcomes. We did not have information regarding cancer stage or treatment and therefore were unable to make statistical adjustments for these differences in our models. We were likely underpowered due to small sample sizes in the race-stratified analysis, and were unable to examine cancer-specific mortality in the present analysis. In particular, given the follow-up time of 5 to 9 years available in this study, less fatal cancer types such as breast and prostate cancer, may have been under-represented in the analysis. However, we plan future studies that include more years of follow-up and a wider range of cancer types to support cancer-specific mortality analysis.

## Conclusions

In conclusion, Black participants with MetS at baseline were at higher risk for cancer mortality during follow-up compared with those without MetS.

## Additional files

Additional file 1: Appendix A: Cancer types among 997 participants with cancer deaths in the REGARDS cohort. (DOCX 45 kb)

Additional file 2: Appendix B: Hazard ratios (HRs)a and 95% confidence intervals for the association between metabolic syndrome (MetS) and cancer mortality (Excluding participants with baseline chronic medical conditions) (DOCX 100 kb)

Additional file 3: Appendix C: Rotated factor loadings for 15 metabolism-associated variables in the REGARDS cohort. (DOCX 71 kb)

Additional file 4: Appendix D: Baseline characteristics of participants by quartiles of derived metabolic factors, REGARDS. (DOCX 107 kb)

Additional file 5: Appendix E: Kaplan-Meier plot for time to cancer death by number of metabolic factor components in the 4th quartile. (DOCX 191 kb)

Additional file 6: Appendix F: Hazard ratios (HRs) a and 95% confidence intervals (CIs) for the association between metabolic dysregulation factors and cancer mortality (Excluding participants with baseline medical conditions). (DOCX 116 kb)

## Abbreviations

AHR: Adjusted hazard ratio
ANOVA: Analysis of variance
BMI: Body mass index
CI: Confidence interval
cm: centimeter
HDL: High-density lipoprotein
HRs: Hazard ratios
hsCRP: high-sensitivity C-reactive protein
kg/m^2^: Kilograms per meter squared
LDL: Low-density lipoprotein
MetS: Metabolic syndrome
mg/dL: Milligrams per deciliter
mmHg: Millimeter of mercury
NDI: National death index
REGARDS: REasons for geographic and racial differences in stroke
SSDI: Social Security Death Index
US: United States
WC: Waist circumference
