# Supplementary material for: Metabolic dysregulation and cancer mortality in a national cohort of blacks and whites
Source: BMC Cancer. 2017 Dec 15;17:856. doi: 10.1186/s12885-017-3807-2 (PMC5731092; doi:10.1186/s12885-017-3807-2)
Supplement: Supplementary file 1 — Appendix A: Cancer types among 997 participants with cancer deaths in the REGARDS cohort. (DOCX 45 kb) [file 12885_2017_3807_MOESM1_ESM.docx]

| **Appendix A. Cancer types among 997 participants with cancer deaths in the REGARDS cohort.** | |
| --- | --- |
|  | **N (%)** |
| Total | 997 |
| **Cancer Type** |  |
| Lung | 274 (27.5) |
| Gastro-intestinal | 205 (20.6) |
| Hematological | 106 (10.6) |
| Genitourinary | 44 (4.4) |
| Prostate | 65 (6.5) |
| Gynecologic | 23 (2.3) |
| Central nervous system | 23 (2.3) |
| Head and neck | 15 (1.5) |
| Breast | 34 (3.4) |
| Other/Unknown | 208 (20.9) |
