# Supplementary material for: Metabolic dysregulation and cancer mortality in a national cohort of blacks and whites
Source: BMC Cancer. 2017 Dec 15;17:856. doi: 10.1186/s12885-017-3807-2 (PMC5731092; doi:10.1186/s12885-017-3807-2)
Supplement: Supplementary file 2 — Appendix B: Hazard ratios (HRs)a and 95% confidence intervals for the association between metabolic syndrome (MetS) and cancer mortality (Excluding participants with baseline chronic medical conditions) (DOCX 100 kb) [file 12885_2017_3807_MOESM2_ESM.docx]

| **Appendix B: Hazard ratios (HRs)^a^ and 95% confidence intervals for the association between metabolic syndrome (MetS) and cancer mortality (Excluding participants with baseline chronic medical conditions)** | | | | |
| --- | --- | --- | --- | --- |
|  | HR (95% CI) | | | |
|  | Black  (*N* = 185)^b^ | White  (*N* = 216)^b^ | All  (*N* = 997)^b^ | *p* value_interaction_^c^ |
| **Metabolic Syndrome** | 1.12 (0.91 – 1.37) | 1.12 (0.95 – 1.33) | 1.12 (0.99 – 1.28) | 0.28 |
| **Components** |  |  |  |  |
| **High WC** | 1.03 (0.83 – 1.28) | 1.03 (0.87 – 1.22) | 1.04 (0.91 – 1.19) | 0.07 |
| **Elevated Triglycerides** | 0.90 (0.69 – 1.19) | 1.03 (0.87 – 1.23) | 0.97 (0.84 – 1.12) | 0.91 |
| **Reduced HDL Cholesterol** | 1.14 (0.92 – 1.40) | 1.15 (0.97 – 1.36) | 1.14 (1.00 – 1.30) | 0.27 |
| **Elevated blood pressure** | 1.19 (0.89 – 1.59) | 0.98 (0.82 – 1.17) | 1.06 (0.91 – 1.23) | 0.89 |
| **Elevated fasting glucose** | 1.21 (0.99 – 1.48) | 1.06 (0.90 – 1.26) | 1.13 (0.99 – 1.29) | 0.57 |
| **# Metabolic Syndrome Components** |  |  |  |  |
| 0 (Referent) | Referent | Referent | Referent | 0.98 |
| 1 | 1.17 (0.70 – 1.96) | 0.99 (0.73 – 1.33) | 1.04 (0.81 – 1.35) |  |
| 2 | 1.19 (0.72 – 1.97) | 0.93 (0.68 – 1.25) | 1.02 (0.80 – 1.32) |  |
| 3 | 1.24 (0.75 – 2.05) | 1.06 (0.78 – 1.44) | 1.12 (0.87 – 1.45) |  |
| 4 | 1.37 (0.80 – 2.33) | 1.10 (0.79 – 1.53) | 1.19 (0.90 – 1.57) |  |
| 5 | 1.50 (0.77 – 2.93) | 1.09 (0.72 – 1.64) | 1.19 (0.84 – 1.68) |  |
| ^a^ Analysis based on 25,038 REGARDS participants with non-missing data on exposure and covariates. Models adjusted for age, sex, race (all model only), education, region, income, tobacco and alcohol use.  ^b^ *N =* number of cancer death events  ^c^ Interaction significance between race*factor (i.e., metabolic syndrome, high waist circumference (WC), and metabolic syndrome components) using Wald test.  **Bold** indicates statistically significant at 0.05 alpha level. | | | | |
