# Supplementary material for: Metabolic dysregulation and cancer mortality in a national cohort of blacks and whites
Source: BMC Cancer. 2017 Dec 15;17:856. doi: 10.1186/s12885-017-3807-2 (PMC5731092; doi:10.1186/s12885-017-3807-2)
Supplement: Supplementary file 3 — Appendix C: Rotated factor loadings for 15 metabolism-associated variables in the REGARDS cohort. (DOCX 71 kb) [file 12885_2017_3807_MOESM3_ESM.docx]

| **Appendix C. Rotated factor loadings for 15 metabolism-associated variables in the REGARDS cohort.** | | | | | | |
| --- | --- | --- | --- | --- | --- | --- |
|  | **Factor 1**  **[Obesity]** | **Factor 2**  **[Cholesterol]** | **Factor 3**  **(Blood Pressure)** | **Factor 4**  **(Lipids)** | **Factor 5**  **(Height)** | **Factor 6**  **(Glucose)** |
| **% Variance explained**^†^ | 25.4 | 13.6 | 11.4 | 9.2 | 8.4 | 6.9 |
| **Factor variables*** | |  |  |  |  |  |
| Weight (kg) | 0.91 | - | - | - | - | - |
| Height (in.) | - | - | - | - | 0.93 | - |
| Waist Circumference (cm) | 0.86 | - | - | - | - | - |
| BMI^†^ | 0.94 | - | - | - | - | - |
| Log Triglycerides (mg/dL) | - | - | - | 0.76 | - | - |
| Total Cholesterol (mg/dL) | - | 0.97 | - | - | - | - |
| HDL-Cholesterol (mg/dL) | - | - | - | -0.69 | - | - |
| Log LDL-Cholesterol (mg/dL) | - | 0.94 | - | - | - | - |
| Dyslipidemia (%) | - | - | - | 0.70 | - | - |
| DBP (mmHg) | - | - | 0.88 | - | - | - |
| SBP (mmHg) | - | - | 0.80 | - | - | - |
| Hypertension (%) | - | - | 0.68 | - | - | - |
| Log Insulin (uU/mL) | 0.61 | - | - | - | - | - |
| Fasting glucose (mg/dL) | - | - | - | - | - | 0.70 |
| Diabetes (%) | - | - | - | - | - | 0.83 |
| ^†^ The percentage of the total variance accounted for by each factor.  *Showing variables with absolute values for factor loadings >0.40.  Analysis based on 19, 963 REGARDS participants with non-missing data on all factor analysis component variables | | | | | | |
