# Supplementary material for: Metabolic dysregulation and cancer mortality in a national cohort of blacks and whites
Source: BMC Cancer. 2017 Dec 15;17:856. doi: 10.1186/s12885-017-3807-2 (PMC5731092; doi:10.1186/s12885-017-3807-2)
Supplement: Supplementary file 4 — Appendix D: Baseline characteristics of participants by quartiles of derived metabolic factors, REGARDS. (DOCX 107 kb) [file 12885_2017_3807_MOESM4_ESM.docx]

| **Appendix D. Baseline characteristics of participants by quartiles of derived metabolic factors, REGARDS** | | | | | | |
| --- | --- | --- | --- | --- | --- | --- |
|  | **Characteristic** | **1^st^ Quartile** | **2^nd^ Quartile** | **3^rd^ Quartile** | **4^th^ Quartile** | ***p* value*** |
| **Factor 1-**  **Obesity** | Mean score^§†^ | -1.14 (0.36) | -0.40 (0.16) | 0.20 (0.20) | 1.34 (0.71) |  |
|  | % Female | 30.4 | 23.1 | 21.5 | 25.0 | <0.001 |
|  | % Black | 18.8 | 22.7 | 25.1 | 33.4 | <0.001 |
|  | % < High School | 24.4 | 23.0 | 23.9 | 28.8 | <0.001 |
|  | % HH Income < $20,000 | 27.3 | 22.2 | 21.9 | 28.7 | <0.001 |
| **Factor 2 -**  **Cholesterol** | Mean score^†^ | -1.26 (0.52) | -0.32 (0.19) | 0.31 (0.19) | 1.26 (0.54) |  |
|  | % Female | 20.6 | 24.3 | 26.2 | 29.0 | <0.001 |
|  | % Black | 23.0 | 24.3 | 25.9 | 26.9 | <0.001 |
|  | % < High School | 28.2 | 23.2 | 24.6 | 24.0 | <0.001 |
|  | % HH Income < $20,000 | 24.9 | 24.9 | 23.8 | 26.4 | <0.001 |
| **Factor 3 -**  **Blood Pressure** | Mean score^†^ | -1.20 (0.39) | -0.39 (0.17) | 0.24 (0.22) | 1.34 (0.59) |  |
|  | % Female | 26.6 | 24.6 | 25.0 | 23.8 | <0.001 |
|  | % Black | 17.0 | 22.6 | 27.3 | 33.1 | <0.001 |
|  | % < High School | 18.6 | 21.2 | 27.9 | 32.3 | <0.001 |
|  | % HH Income < $20,000 | 20.6 | 21.3 | 25.7 | 32.4 | <0.001 |
| **Factor 4 -**  **Lipids** | Mean score^†^ | -1.28 (0.43) | -0.37 (0.21) | 0.37 (0.22) | 1.28 (0.39) |  |
|  | % Female | 29.9 | 28.1 | 22.8 | 19.3 | <0.001 |
|  | % Black | 32,9 | 27.8 | 23.9 | 15.5 | <0.001 |
|  | % < High School | 22.4 | 26.0 | 24.5 | 27.1 | <0.001 |
|  | % HH Income < $20,000 | 23.9 | 26.6 | 23.8 | 25.7 | <0.001 |
| **Factor 5 -**  **Height** | Mean score^†^ | -1.25 (0.40) | -0.40 (0.20) | 0.33 (0.23) | 1.31 (0.45) |  |
|  | % Female | 43.4 | 37.2 | 16.9 | 2.5 | <0.001 |
|  | % Black | 27.8 | 26.9 | 24.7 | 20.7 | <0.001 |
|  | % < High School | 30.1 | 26.2 | 24.0 | 19.8 | <0.001 |
|  | % HH Income < $20,000 | 34.9 | 28.3 | 22.4 | 14.5 | <0.001 |
| **Factor 6 -**  **Glucose** | Mean score^†^ | -0.65 (0.18) | -0.34 (0.06) | -0.12 (0.07) | 1.11 (1.48) |  |
|  | % Female | 24.8 | 25.1 | 25.5 | 24.6 | 0.2 |
|  | % Black | 25.7 | 22.2 | 23.5 | 28.7 | <0.001 |
|  | % < High School | 26.4 | 22.7 | 21.8 | 29.1 | <0.001 |
|  | % HH Income < $20,000 | 24.9 | 22.6 | 22.5 | 30.0 | <0.001 |
| ^†^Presented as mean (standard deviation).  *Significance determined using Chi-Square test.  Household = HH  ^§^Mean factor score derived from factor analysis. | | | | | | |
