# Supplementary material for: Metabolic dysregulation and cancer mortality in a national cohort of blacks and whites
Source: BMC Cancer. 2017 Dec 15;17:856. doi: 10.1186/s12885-017-3807-2 (PMC5731092; doi:10.1186/s12885-017-3807-2)
Supplement: Supplementary file 5 — Appendix E: Kaplan-Meier plot for time to cancer death by number of metabolic factor components in the 4th quartile. (DOCX 191 kb) [file 12885_2017_3807_MOESM5_ESM.docx]

| **Appendix E: Kaplan-Meier plot for time to cancer death by number of metabolic factor components in the 4^th^ quartile.** |
| --- |
|  |
