# Supplementary material for: Metabolic dysregulation and cancer mortality in a national cohort of blacks and whites
Source: BMC Cancer. 2017 Dec 15;17:856. doi: 10.1186/s12885-017-3807-2 (PMC5731092; doi:10.1186/s12885-017-3807-2)
Supplement: Supplementary file 6 — Appendix F: Hazard ratios (HRs) a and 95% confidence intervals (CIs) for the association between metabolic dysregulation factors and cancer mortality (Excluding participants with baseline medical conditions). (DOCX 116 kb) [file 12885_2017_3807_MOESM6_ESM.docx]

| **Appendix F: Hazard ratios (HRs)** ^a^ **and 95% confidence intervals (CIs) for the association between metabolic dysregulation factors and cancer mortality (Excluding participants with baseline medical conditions)** | | | | |
| --- | --- | --- | --- | --- |
|  | Black  (*N* = 287) ^b^ | White  (*N* = 497) ^b^ | All  (*N* = 784) ^b^ | *p* value_interaction_ ^c^ |
| **Obesity** |  |  |  |  |
| 1^st^ Quartile (Ref) | Referent | Referent | Referent | 0.25 |
| 2^nd^ Quartile | 0.72 (0.51 – 1.01) | 0.83 (0.65 – 1.05) | **0.79 (0.65 – 0.96)** |  |
| 3^rd^ Quartile | **0.64 (0.45 – 0.91)** | **0.76 (0.59 – 0.98)** | **0.72 (0.59 – 0.88)** |  |
| 4^th^ Quartile | 0.77 (0.56 – 1.08) | 0.77 (0.58 – 1.02) | **0.78 (0.64 – 0.97)** |  |
| **Cholesterol** |  |  |  |  |
| 1^st^ Quartile (Ref) | Referent | Referent | Referent | 0.27 |
| 2^nd^ Quartile | 0.91 (0.66 – 1.25) | **0.67 (0.52 – 0.85)** | **0.75 (0.62 – 0.91)** |  |
| 3^rd^ Quartile | 0.76 (0.54 – 1.07) | 0.79 (0.62 – 1.01) | **0.78 (0.64 – 0.95)** |  |
| 4^th^ Quartile | 0.81 (0.58 – 1.13) | **0.77 (0.60 – 0.99)** | **0.78 (0.64 – 0.96)** |  |
| **Blood Pressure** |  |  |  |  |
| 1^st^ Quartile (Ref) | Referent | Referent | Referent | 0.55 |
| 2^nd^ Quartile | 1.14 (0.77 – 1.68) | 1.06 (0.83 – 1.36) | 1.09 (0.89 – 1.34) |  |
| 3^rd^ Quartile | 1.00 (0.68 – 1.46) | 0.95 (0.74 – 1.23) | 0.98 (0.79 – 1.21) |  |
| 4^th^ Quartile | 0.96 (0.66 – 1.39) | 1.07 (0.83 – 1.38) | 1.02 (0.83 – 1.26) |  |
| **Lipids** |  |  |  |  |
| 1^st^ Quartile (Ref) | Referent | Referent | Referent | 0.55 |
| 2^nd^ Quartile | 1.12 (0.82 – 1.52) | 0.93 (0.70 – 1.25) | 1.01 (0.82 – 1.24) |  |
| 3^rd^ Quartile | 0.84 (0.60 – 1.18) | 0.89 (0.67 – 1.19) | 0.87 (0.70 – 1.09) |  |
| 4^th^ Quartile | 0.96 (0.67 – 1.37) | 1.06 (0.81 – 1.38) | 1.03 (0.84 – 1.27) |  |
| **Height** |  |  |  |  |
| 1^st^ Quartile (Ref) | Referent | Referent | Referent | 0.07 |
| 2^nd^ Quartile | 0.83 (0.57 – 1.21) | 0.94 (0.71 – 1.26) | 0.90 (0.72 – 1.14) |  |
| 3^rd^ Quartile | 1.22 (0.83 – 1.81) | 0.78 (0.56 – 1.09) | 0.94 (0.73 – 1.21) |  |
| 4^th^ Quartile | 0.83 (0.52 – 1.31) | 0.73 (0.51 – 1.05) | 0.79 (0.59 – 1.04) |  |
| **Glucose** |  |  |  |  |
| 1^st^ Quartile (Ref) | Referent | Referent | Referent | 0.58 |
| 2^nd^ Quartile | 1.24 (0.86 – 1.78) | 1.23 (0.94 – 1.60) | **1.24 (1.01 – 1.54)** |  |
| 3^rd^ Quartile | 1.16 (0.80 – 1.66) | 1.30 (0.99 – 1.69) | **1.26 (1.02 – 1.56)** |  |
| 4^th^ Quartile | **1.48 (1.07 – 2.05)** | 1.28 (0.97 – 1.67) | **1.37 (1.11 – 1.69)** |  |
| **# Factor Variables in 4^th^ Quartile** |  |  |  |  |
| 0 (Referent) | Referent | Referent | Referent | 0.08 |
| 1 | 0.75 (0.52 – 1.08) | 1.30 (0.98 – 1.73) | 1.07 (0.85 – 1.34) |  |
| 2 | 0.83 (0.58 – 1.18) | 1.29 (0.96 – 1.73) | 1.09 (0.87 – 1.37) |  |
| 3+ | 0.87 (0.58 – 1.31) | 1.09 (0.78 – 1.54) | 1.01 (0.78 – 1.31) |  |
| ^a^ Analysis based on 19, 963 REGARDS participants with non-missing data on all factor analysis component variables. Models adjusted for age, sex, race (all model only), education, region, income, tobacco and alcohol use.  ^b^ *N =* number of cancer death events.  ^c^ Interaction significance between race*factor using Wald test. **Bold** indicates statistically significant at 0.05 alpha level. | | | | |
